# Antimicrobial efficacy of sodium hypochlorite and hyper-pure chlorine dioxide in the depth of dentin tubules in vitro

**DOI:** 10.1186/s12903-023-03685-6

**Published:** 2023-11-27

**Authors:** Enikő Vasziné Szabó, Brigitta Huszta, Melinda Polyák, Kasidid Ruksakiet, Róbert Bernáth, Ágoston Ghidán, Ágnes Csáki, Milia Kostadinova, Elek Dinya, János Vág, Zsolt M. Lohinai

**Affiliations:** 1 Department of Restorative Dentistry and Endodontics, Semmelweis University, Szentkirály u. 47, H-1088 Budapest, Hungary; 2 Department of Oral Biology, Semmelweis University, Nagyvárad tér 4, H-1089 Budapest, Hungary; 3 Department of Oral Diagnostics, Semmelweis University, Nagyvárad tér 4, H-1089 Budapest, Hungary; 4 Department of Medical Microbiology, Semmelweis University, Nagyvárad tér 4, H-1089 Budapest, Hungary; 5 Department of Anatomy, Histology and Embryology, Semmelweis University, Tűzoltó u. 58, H-1094 Budapest, Hungary; 6 Institute of Digital Health Sciences Semmelweis University, Ferenc tér 15, H-1094 Budapest, Hungary; 7 Department of Restorative Dentistry, Faculty of Dentistry, Naresuan University, Tha Pho, Mueang Phitsanulok District, 65000 Phitsanulok, Thailand

**Keywords:** Confocal laser scanning microscopy, Disinfection, Chlorine dioxide, Sodium hypochlorite, Antimicrobial effectiveness, *Enterococcus faecalis*

## Abstract

**Objectives:**

The study aimed to compare the antibacterial effect of a novel disinfectant, hyper-pure chlorine dioxide (hClO_2_) to sodium hypochlorite (NaOCl) in various depths of dentin tubules.

**Materials and methods:**

The distal root of the extracted lower molars was infected artificially with *Enterococcus faecalis*. The control group was rinsed with saline, and the test groups were irrigated with either 5% NaOCl or 0.12% hClO_2_. The longitudinally split teeth were stained by viability stain. The coronal third of the root was scanned with a confocal laser scanning microscope. The fluorescent intensities were measured, and the percentage of dead bacteria was calculated at depths up to 950 μm along the dentin tubules. The effect of penetration depth, irrigants, and their interaction on antimicrobial efficacy was determined by the linear mixed model.

**Results:**

The percentage of dead bacteria was higher both in the NaOCl (45.1 ± 2.3%, p < 0.01) and in the hClO_2_ (44.6 ± 3.8%, p < 0.01) irrigant groups compared to saline (23 ± 4.5%); however, there was no difference between them. The percentage of killed bacteria was not correlated with the depths in any group (p = 0.633).

**Conclusions:**

Our results suggest that the functional penetration depth of NaOCl is at least 2–3 times more than published to date. There is no difference in disinfection effectiveness along the dentin tubules between NaOCl and hClO_2_ until at least the measured 950 μm. However, both were only able to eradicate the intratubular bacteria partially.

**Clinical relevance:**

Hyper-pure ClO_2_ could be used as an alternative or final adjuvant irrigant in endodontic treatment.

**Supplementary Information:**

The online version contains supplementary material available at 10.1186/s12903-023-03685-6.

## Introduction

Root canal treatment of infected teeth aims to completely eradicate pathogenic microorganisms embedding and penetrating the root canal system before the final obturation. Mechanical preparation alone has proven to be inefficient due to the complexity of the root canal system [1]. Consequently, the significance of complementary chemical antisepsis becomes more critical. Most failures of endodontic treatments occur because of the ineffective elimination of pathogenic microorganisms [2], even if root canal obturation seems faultless. About 40% of teeth that are root canal treated are associated with apical inflammation, thereby indicating failure [3]. Even teeth that do not show visible radiographic lesions can still harbor pathogenic microorganisms [4], which may cause periapical pathosis if the host immune system becomes compromised. Bacterial invasion of the root dentin wall can be seen as far as 400 μm by scanning electron microscopy [5], but bacterial endotoxins can be detected even deeper at 1200 μm [3].

*Enterococcus faecalis* (*E. faecalis*) is an opportunistic pathogen in the oral cavity [6]. It is a Gram-positive, facultative anaerobe that is able to survive extreme conditions or starvation [7]. It is resistant to disinfection even as a single organism without being part of a supportive biofilm environment [8]. Four to 40% of primary infections show the presence *E. faecalis*, and 24 to 77% of teeth with persistent infection present cultivable, that is viable *E. faecalis* [9]. Therefore, its elimination is essential for the success of endodontic treatment.

The gold-standard disinfectant irrigant solution in endodontics is still sodium hypochlorite (NaOCl). Commercial products of NaOCl are most commonly around pH 12 showing strong base characteristic. At this pH the solution contains 5 to 12% free available chlorine. These strong oxidizing agents that react with aminoacids, peptides, proteins, lipids, and DNA by attacking the C = C double bonds [10]. NaOCl has a wide spectrum against the different pathogenic microorganisms that cause endodontic infection [11–12]; however, it is highly cytotoxic when extruded over the apical foramen [13–14]. NaOCl has a high surface tension [15], allowing only 300 μm penetration into dentin tubules in vitro according to a study [16]. This distance is far too little to have sufficient disinfection of the tubules. On the other hand, it is known that *E. faecalis* had higher resistance to NaOCl than did other microorganisms [17].

Chlorine dioxide (ClO_2_) has been used as a disinfectant in the food industry and water treatment since 2008 [18]. It has higher oxidation power than NaOCl [19], and has 2,5 times more potential than chlorine [20]. As an “ideal biocide,” it was suggested to be used in endodontics [21]. Because of its extremely small size, it allows easy access into small spaces such as dentin tubules. Novel membrane technology made it possible to produce a hyper-pure aqueous solution of ClO_2_ (hClO_2_) [22] to avoid the disadvantages of the commercially available stabilized ClO_2_ desinfectants. This hyper-pure solution does not contain any acidic by-products from manufacturing as ClO_2_ solutions prepared with other processing technologies. As a true gas solution, it is very volatile [22–23]. It reacts with only three aminoacids, but not with larger molecules. Because of its high volatility the contact time of hClO_2_ is limited. Due to the short contact time and its reactiveness, it could kill a bacterial cells, but not harm human cells, which, on the other hand, also have a natural protection mechanisms such as the antioxidant glutathione [24]. Studies have been conducted by our group to test its effectiveness and safety briefly summarized in the following papers. Because of its mechanism of action, it is a potent antimicrobial agent which was tested in vitro on oral pathogenic microorganisms [25]. In extracted tooth models artificially infected by *E. faecalis*, irrigation with hClO_2_ showed significantly less rebound of bacterial count in the samples after 2 and 5 days of temporization than NaOCl [26]. On the other hand, our cell viability experiments have demonstrated much better biocompatibility on human periodontal ligament stem cells compared to chlorhexidine and hydrogen peroxides [27].

Currently, no method or irrigant solution can achieve total elimination of biofilm from the root canal system. The biocompatibility of the gold-standard NaOCl is also of concern especially if used in regenerative endodontics [28]. Based on our previous results [25–27] on the potential clinical benefit of hClO_2_, we hypothesized that it could exert its antimicrobial efficacy at least as deep as NaOCl along the dentin tubules and at least with the same effectiveness.

We aimed to determine the depth of antimicrobial activity of both NaOCl and hClO_2_ along the dentin tubules and to compare their antimicrobial effectiveness at various depths in an in vitro root segment model.

## Materials and methods

The distal root of 27 extracted lower mandibular molar teeth with no prior endodontic treatment were collected. Patient consent was obtained for the in vitro use of the teeth according to conditions set by the Regional Ethical Committee of Scientific Affairs (SE-RKEB 205/2021). The teeth were stored in saline at 4 °C until use.

### Root segment model preparation artificially inoculated by *E. faecalis*

The teeth were prepared for inoculation by the modified protocol of Andrade et al. [29]. Briefly, the teeth were stored in 1% NaOCl (Pharmacy of Semmelweis University, Budapest, Hungary) for 48 h. After decoronation the outer cement layer of the roots was removed by a diamond bur (Gallax Dental Kft., Budapest, Hungary) using water cooling to expose the dentin tubules. The roots were mechanically prepared with #40 RECIPROC^®^ blue file (V.D.W. GmbH, Munich, Germany) 1 mm shorter than the apical foramen. In order to open the dentin tubules and to remove smear layers from both endings, they were immersed in 17% EDTA (Cerkamed, Stalowa Wola, Poland) for 1 min. Additionally, it was stimulated by sonic intracanal activation using the EDDY system (V.D.W. GmbH, Munich) for 60 s. Next, the roots were washed with distilled water, dried, and embedded in Eppendorf tubes (Merck, KGaA, Darmstadt, Germany; 1,5 mL). They were subsequently autoclaved at 121 °C for one hour in saline (Fresenius Kabi Hungary Kft., Budapest, Hungary). Three roots were randomly chosen to be kept sterile as the first absolute controls in group 1 (n = 3) (Table 1.). The remaining roots were prepared for inoculation with 3 × 10^8^ CFU/mL *E. faecalis* (ATCC 29,212) centrifugation protocol [29].

**Table 1:** Random division of roots into the five study group. *NA: not applicable

groups		number of samples	inoculation	treatment	time of irrigation	amount of irrigation
group 1	1st absolute control	3	-	-	NA	NA
group 2	2nd absolute control	3	+	-	NA	NA
group 3	saline negative control	7	+	saline	10 min.	4 mL
group 4	NaOCl positive control	7	+	5% NaOCl	10 min.	4 mL
group 5	hClO_2_	7	+	0.12% hClO_2_	10 min.	4 mL

### Treatment protocol

After four days of incubation, completed with a centrifugation procedure, the culturing BHI medium (Mast Group Ltd., Merseyside, U.K.) was removed. The remaining medium was washed out with 2 mL saline. The dentin tubules and the apical foramen were sealed from the outside of the root by covering the outer surface of the roots with two layers of nail polish. We waited two minutes between each layer for the drying of the nail polish. The teeth were randomly divided into four groups. The second absolute control group 2 (n = 3) received no disinfection. Group 3 was irrigated with saline (n = 7), group 4 with 5% NaOCl (n = 7), group 5 with 0.12% hyper-pure ClO_2_ (n = 7, Solumium Dental, lately called Solumium Pental; Solumium Kft., Budapest, Hungary) (Table 1.). Each solution was applied by continuous flushing of 2 mL for 30 s using NaviTip^®^ 29 Gauge (Ultradent Products Inc., South Jordan, UT, U.S.A.) in the depth of working length minus 1 mm. The irrigants were manually activated by #15 K-file (Kerr Co., Kloten, Switzerland) for another 30 s. After eight minutes, the irrigation was repeated. A total of 4 mL irrigant solution was used for each root for an effective time of 10 min. The disinfectant reaction was stopped by a final rinse with 2 mL saline.

### Confocal laser scanning microscopy (CLSM) imaging

A groove was prepared by a diamond disc along the axis of the root on the mesial side until the root canal. A blade was fit in the groove, then the roots were longitudinally split with a hammer and were mounted on glass slides. The split surface to be stained and examined was cleaned with 17% EDTA for 10 min to remove the smear layer. The samples were then washed with saline and dried. They were stained with 30 µL of LIVE/DEAD® BacLight_TM_ Viability stain (LIVE/DEAD® BacLight_TM_ Viability stain L7007, Invitrogen Molecular Probes, Eugene, OR, U.S.A.) for 20 min according to the protocol of Andrade et al. [29]. The viability stain in a 1:1 ratio of components A and B was applied just prior to scanning. The roots were examined under Zeiss. Imager. Z2 LSM 780 (Carl Zeiss MicroImaging GmbH, 07740 Jena, Germany) confocal microscope. Live bacteria were seen in green fluorescence stained by SYTO9. Dead bacteria were fluorescent in red after staining with Propidium Iodide (PI). The distal split surfaces were scanned at 10x objective magnification using a frame size of 1024 × 1024 pixels. The pixel size was 1,38 μm. Twenty-one slices were scanned with 10 μm step size using a filter range of 500–590 nm for SYTO9 and 560–680 nm for PI [29]. The scan was performed in the coronal third of the root canal. The images were acquired by the Zeiss Zen 212 SPS program (Carl Zeiss Microscopy Deutschland GmbH, Carl Zeiss Strasse 22 73,447 Oberkochen, Deutschland).

**Fig. 1:**
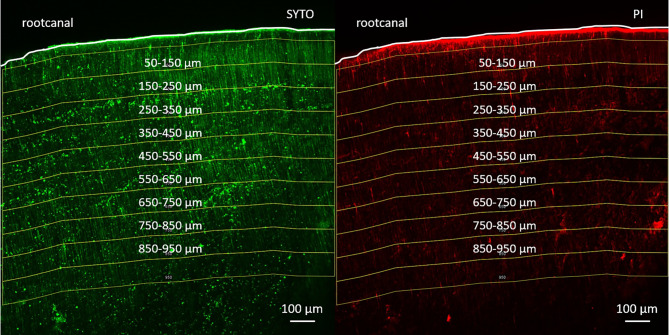
*The distance intervals selected from the root canal surface in a confocal laser scanning microscopy image.* Live bacteria are shown on the left in green after being stained by SYTO9. Dead bacteria are shown on the right in red after being stained by Propidium Iodide (PI). A white line outlines the root canal border

The fluorescence of dead and live bacteria was measured at the following distance intervals starting from 50 μm from the root canal surface: 50–150 μm, 150–250 μm, 250–350 μm, 350–450 μm, 450–550 μm, 550–650 μm, 650–750 μm, 750–850 μm, 850–950 μm (Fig. 1.). Measurement of fluorescence intensity of dead bacteria was done with the Image J program with Fiji extension [30–31]. The effectiveness of the irrigant solutions was calculated by the following equation [32]: dead bacteria % = red intensity / (red intensity + green intensity) x 100.

### Statistical analysis

The primary outcome was to detect a significant decrease in the percentage of viable bacteria in group 5 (0.12% hClO_2_) compared to group 4 (5% NaOCl, gold-standard). The effect size was estimated from the result of a previous study using the same method [33]. In that particular study, a new agent halved the viable cells. We estimated a similar effect for hClO_2_. The required sample size was estimated by the G*power (University of Düsseldorf, Düsseldorf, Germany). The alpha was set to 0.05, and the power was 0.80 (1-β). The calculations revealed that six samples per group were needed to detect a 50% drop in viable cells.

The effect of penetration depth, irrigants, and their interaction on antimicrobial efficacy was determined by the linear mixed model. The penetration depth was continuous, and the irrigant was a categorical fixed factor. The tooth was the random subject factor in the model. The fixed effects represent the mean of the trajectory pooling of all teeth within the sample, and the random effects represent the variance of the individual trajectories around group means. For each irrigant, a linear regression equation was calculated from the model. Statistical significance was set at a p < 0.05. Statistical evaluation was carried out by the SPSS software (I.B.M. Inc., version 28). Values in the text and graph are given as a mean ± 95% confidence interval.

## Results

**Fig. 2:**
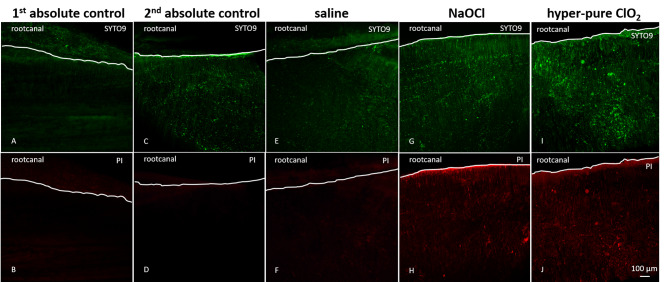
*Representative confocal laser scanning microscopy images of live and dead bacterial staining in the investigated groups.* Samples in the first absolute control group (**A** and **B**) were kept sterile. Samples in the second absolute group (**C** and **D**) were inoculated but not irrigated. Groups with irrigation were a saline group (E and F), a NaOCl group (G and H), and a hClO_2_ (**I** and **J**). The upper row (**A**, **C**, **E**, **G**, **I**) shows SYTO9 staining of live bacteria, fluoresced in green. The lower row (**B**, **D**, **F**, **H**, **J**) shows Propidium Iodide (PI) staining of dead bacteria fluoresced in red. The white line outlines the root canal border

In the first absolute control group, no bacteria were found in the dentin tubules (Fig. 2. A, B). However, the inoculated but not irrigated samples (second absolute control group) showed the presence of live bacteria stained by SYTO9 (Fig. 2. C, D). The representative CLSM pictures of the irrigated groups (saline, NaOCl, and hClO_2_) are shown in Fig. 2. (E-J).

**Fig. 3:**
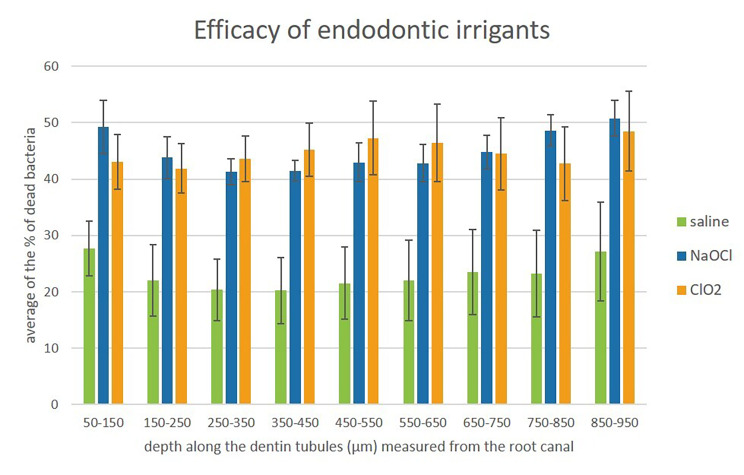
*The penetration depth of irrigant solutions.* Significance was not calculated because the slope of the linear equations showed that the ‘depth’ factor had no influence on the % of dead bacteria

Both disinfectants penetrated dentin tubules at least 950 μm, as seen in the bar graph (Fig. 3.).

**Fig. 4:**
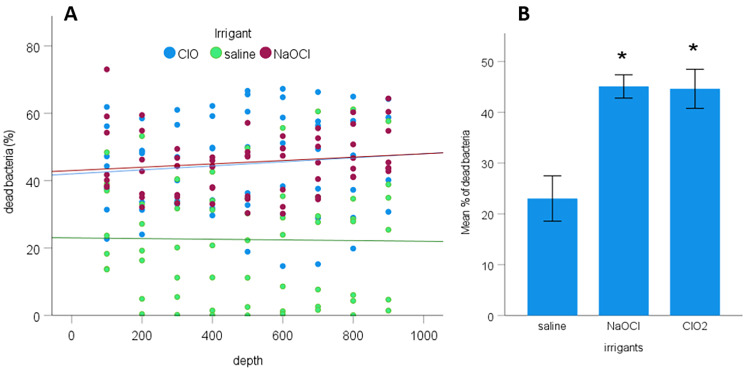
** A**. *The relationship between the penetration depth of irrigants measured from the root canal surface and the percentage of killed bacteria.***B**. *The mean effect of irrigants on the percentage of dead bacteria.* Error bars indicate 95% confidence intervals. Slopes are almost parallel to the x-axis, indicating an insignificant effect of “depth”. The significant difference between the test (NaOCl and hClO_2_) and saline groups are indicated by an asterisk. * p < 0.01

The linear regression equation for each irrigant was calculated from the model. The interaction between penetration depth and irrigants (p = 0.893) and the main effect of penetration depth (p = 0.633) were insignificant. As seen in Fig. 4A., the slopes of the linear equations of all irrigants were almost parallel to the x-axis and showed no correlation between the penetration depth and the irrigant solutions seen in any group (Fig. 4A.). The linear equations between penetration depth and percentage of dead bacteria were the following:

y_NaOCl_ = 42.7 + 0.005x.

y_ClO2_ = 42.2 + 0.006x.

y_saline_ = 23.2–0.001x.

The overall antibacterial effect of NaOCl (45.1 ± 2.3%, p < 0.01) and hClO_2_ (44.6 ± 3.8%, p < 0.01) was significantly higher than that of saline (23 ± 4.5%). No significant difference was found between the overall effectiveness of NaOCl and hClO_2_ (Fig. 4B.).

## Discussion

The basic requirements of endodontic irrigant solutions are (1) to be able to penetrate into the site to be disinfected, (2) to suppress the growth of pathogenic microorganisms, (3) to have an effective concentration on pathogenic microorganisms, but at the same time to be non-toxic to human cells and (4) to disinfect without pathogenic microorganisms developing resistance against it [15]. Hyper-pure ClO_2_ seems to fulfill these criteria.

In the root segment model, the dentin tubules were inoculated by *E. faecalis* successfully at least 950 μm deep. Furthermore, the NaOCl and hClO_2_ killed bacteria at the measured 850–950 μm. Contrarily, a previous study using a dye bleaching technique showed 300 μm diffusion of NaOCl along the dentin tubules [16]. Other studies [33, 35-] demonstrated NaOCl antimicrobial activity at a depth of 150 or 500 μm with the similar CLSM method used in this study. Therefore, our results show more than 2–3 times the depth as published before [16, 31–32] (for details, see Table 1 in the Appendix), making it an auspicious outcome in the research regarding the antimicrobial activity of endodontic irrigants in the depth of dentin. It is even more convincing considering that bacterial invasion of root dentin under natural conditions could be seen as far as 400 μm by scanning electron microscopy [5]. Whereas, bacterial endotoxins could be detected as deep as 1200 μm [34]. Furthermore, in our study, the effectiveness of the irrigants did not decline at 950 μm, indicating that the penetration of the irrigants may continue further. However, an investigation into deeper dentin is necessary to prove it.

The clinically relevant concentration of hClO_2_ is only 0.12%, which is an order of magnitude less than that of 2.5-5% NaOCl. In an in vitro study, after irrigation and temporization of the roots, it was able to reduce the regrowth of bacteria after both 2 and 5 days following disinfection [26], showing that it is able not only to eliminate bacteria but to suppress their growth. Previous studies [25, 26] indicated that hClO_2_ was much more effective on *E. faecalis* than NaOCl due to its gas and redissolved phases.

The effective antibacterial concentration in endodontics showed no toxic effect on human periodontal ligamental stem cells compared to NaOCl [27]. Nevertheless, in this study, hClO_2_ was still similarly effective as NaOCl on *E. faecalis* in the same depth of dentin tubules, even in a smaller concentration. Both disinfectants were statistically more effective than saline, but unfortunately, they could only partially eradicate the intratubular bacteria. Considering the natural background amount of dead bacteria seen by irrigation with saline, the effective antibacterial effect of both tested irrigants was around 22%. A stronger antimicrobial effect of both irrigants was expected. The inability of hClO_2_ to interact with other irrigant solutions [35], its higher biocompatibility [27], and its lower effective clinical concentration [25–26, 36] suggest it as an alternative or adjuvant final endodontic irrigant.

The patency of the dentin tubules of the pre-sterilized teeth used in the artificially infected model showed significant heterogeneity in bacterial presence. Both ends of the tubules were opened up to facilitate the inoculation of *E. faecalis* by centrifugation. Previously, the unsatisfactory and heterogeneous inoculation resulted in poor reproducibility; therefore, several modifications have been made since its introduction [29, 37]. The success of the artificial bacterial inoculation does not only depend on the technique but greatly depends on the age of the patient [38], the dental history of the tooth [39–40], and systemic factors (e.g.: hypercalcemia, cardiovascular diseases, glucocorticoid treatment) as well [41–43]. These factors may induce tubular sclerosis, hindering the patency of the tubules [44]. The limit of this study was that the complete dental history of the tooth, including the factors mentioned above, was unknown. Physiological sclerosis starts in the third decade of life, starting apically and moving coronally from the outer surface of the root towards the root canal [39, 45]. Sclerosis shows a typical pattern called the “butterfly effect”, which occurs more intensely in the mesio-distal direction [44–45]. A higher amount of sclerotized dentin in the distal area could have contributed to the inhomogeneous artificial infection and disinfection (see Figs. 2 and 3) in our model. Dentinal sclerosis, however, should not affect the live/dead ratio of bacteria in the tubules as neither NaOCl nor hClO_2_ react with inorganic dental tissues, thereby preventing a decrease in their gradient [15, 46].

## Conclusion

The functional penetration depth of NaOCl along dentin tubules is at least 2–3 times more than what has been published to date. There is no difference in disinfection effectiveness along the dentin tubules between NaOCl and hClO_2_ until at least the measured 950 μm, but both were unfortunately only able to eradicate the intratubular bacteria partially. Hyper-pure ClO_2_ was similarly effective as NaOCl, but in ten times less concentration, therefore, due to its extremely low toxicity, it is suggested as a final irrigant after smear layer removal.

### Electronic supplementary material

Below is the link to the electronic supplementary material.

Supplementary Material 1

## Data Availability

The datasets used and/or analysed during the current study available from the corresponding author on reasonable request.
