# Supplementary material for: Antimicrobial efficacy of sodium hypochlorite and hyper-pure chlorine dioxide in the depth of dentin tubules in vitro
Source: BMC Oral Health. 2023 Nov 27;23:930. doi: 10.1186/s12903-023-03685-6 (PMC10680282; doi:10.1186/s12903-023-03685-6)
Supplement: Supplementary file 1 — Supplementary Material 1 [file 12903_2023_3685_MOESM1_ESM.pdf]

Appendix

see in first paragraph of Discussion

Appendix Table 1 *Study details from the literature using viability staining of bacteria in dentin tubules of root by CLSM method compared to our investigation* [28, 31, 32, 44-46]. Ag np: silver nanoparticles, DW: destilled water, PIPS: photoninduced photoacoustic streaming, US: ultrasonic bath.

Author	Type of tooth	Sterilization of tooth before inoculation	Sample type	Root canal enlargement	Method of inoculation	Steps of study	No. of specimens in each group	Treatment method	Study groups	Staining protocol	Wavelengths of emmission	Magnification	Root segment scanned	Measured parameter	Depth of penetration
Ma et al., 2011	not known<br>single rooted	0,01% NaOCl hrs. not given<br>preparation of root block<br>enlargement of root canal<br>US bath:<br>5,25% NaOCl 4 min.<br>6% citric acid 4 min.	4mm block<br>(apex removed)	#6<br>GatesGlidden	1st day centrifugation of inoculum, incubation<br>2nd day CLSM	1. splitting sample<br>2. inoculation<br>3. treatment<br>4. staining	n=2	drop of disinfectant on inner surface of split sample	1% NaOCl 1 min<br>1% NaOCl 3 min<br>2% NaOCl 1 min<br>2% NaOCl 3 min<br>6% NaOCl 1 min<br>6% NaOCl 3 min<br>CHX 1 min<br>CHX 3 min<br>Qmix 1 min	LIVE/DEAD BacLight viability staining according to manufacturer's instructions<br>µL and min. not given	500 nm SYTO9<br>635 nm PI	20x	coronal	volume of percentage of red fluorescence in three-dimensional model of scanned area	measured until 500 µm
Andrade et al., 2015	bovine	1% NaOCl 48hrs.<br>preparation of root block<br>enlargement of root canal<br>17% EDTA 5 min. in root canal<br>nail polish on outer surface<br>autoclave 121°C, min. not given	12mm block<br>(apical 5mm removed)	#120<br>Kerr-file	1st day centrifugation of inoculum, incubation<br>2nd day centrifugation of BHI, incubation<br>3rd day centrifugation of inoculum, incubation<br>4th day centrifugation of BHI, incubation<br>5th day CLSM	1. inoculation<br>2. cutting sample<br>3. staining<br>no treatment, methodology	n=5	NA	NA	17% EDTA 10 min.<br>LIVE/DEAD BacLight viability staining:<br>30 µL 20 min.	not given	40x	coronal, middle	volume of green and red fluorescence in three-dimensional model of scanned area	not measured
Azim et al., 2016	human mandibular premolar	enlargement of root canal<br>autoclave 121°C, 20 min.	size of root not given	#25/04<br>EndoSeq	Ma et al. 2011 protocol	1. inoculation<br>2. treatment<br>3. splitting sample<br>4. staining	n=4	irrigation or root canal with<br>2 mL 17% EDTA 1 min and<br>3 mL 6% NaOCl 2 min with activation	activation methods:<br>PIPS<br>hand<br>US<br>XP Endo	LIVE/DEAD BacLight viability staining according to manufacturer's instructions<br>15 µL, min. not given	500 nm SYTO9<br>635 nm PI	20x	coronal, middle, apical	percentage of red fluorescence in distances:<br>50 µm, 100 µm, 150 µm	measured until 150 µm
Cavenago et al., 2017	bovine single rooted	preparation of root block<br>enlargement of root canal<br>immerse:<br>2,5% NaOCl 5 min.,<br>17% EDTA 5 min.<br>nail polish on outer surface<br>autoclave 121°C, min. not given	3mm block<br>(apex removed)	#120<br>Kerr-file	Andrade et al. 2015 protocol	1. inoculation<br>2. treatment<br>3. cutting sample<br>4. staining	not well defined	root canal filled with test material	MTA + DW<br>MTA + propylene glycol<br>MTA + Arcticum lappa L.<br>MTA + Casearia sylvestris	17% EDTA 5 min.<br>LIVE/DEAD BacLight viability staining:<br>30 µL 10 min.	not given	40x	apical	percentage of green and red fluorescence in scanned area	not measured
Rodrigues et al., 2018	bovine single rooted	1% NaOCl 48 hrs.<br>preparation of root block<br>enlargement of root canal<br>US bath:<br>1% NaOCl 10 min.,<br>17 % EDTA 10 min.<br>nail polish on outer surface<br>autoclave 121°C, min. not given	12mm block<br>(apical 5mm removed)	#120<br>Kerr-file	Andrade et al. 2015 protocol	1. inoculation<br>2. treatment<br>3. splitting sample<br>4. staining	n=8	irrigation of root canal with disinfectant	1 mL ,5% NaOCl +<br>Na thiosulphate<br>1 mL CHX<br>1 mL Ag np	17% EDTA 5 min.<br>LIVE/DEAD BacLight viability staining:<br>30 µL 20 min.	490-575 nm SYTO9<br>600-720 nm PI	40x	coronal, middle	percentage of green fluorescence in scanned area	not measured
Giardino et al., 2019	human single rooted	preparation of root block<br>enlargement of root canal<br>US bath:<br>5% NaOCl 5 min.,<br>17% EDTA 5 min.<br>nail polish on outer surface<br>autoclave 121°C, min. not given	8mm block<br>(apical 5 mm removed)	#30<br>ProTaper Next	Andrade et al. 2015 protocol	1. inoculation<br>2. treatment<br>3. cutting sample<br>4. staining	n=10	irrigation of root canal with disinfectant	5ml 5% NaOCl 5 min +<br>2ml 17% EDTA 2 min<br>5ml 5% NaOCl +<br>DualRinse HEDP 5 min	LIVE/DEAD BacLight viability staining:<br>30 µL 20 min.	490-575 nm SYTO9<br>600-720 nm PI	40x	coronal, middle	percentage of green fluorescence in scanned area	not measured
This study	human mandibular molar<br>D root	1% NaOCl 48 hrs.<br>length of root adjusted<br>remove outer cementum<br>enlargement of root canal<br>17% EDTA 1 min. in root canal, activation by EDDY system<br>immerse: 17% EDTA 5 min.<br>autoclave 121°C 20 min.	11mm root w/ apex	#40<br>Reciproc Blue	Andrade et al. 2015 protocol	1.inoculation<br>2. treatment<br>3. splitting sample<br>4. staining	n=7	irrigation of root canal with disinfectant	4 mL saline 10 min<br>4 mL 5% NaOCl 10 min<br>4 mL 0,12% ClO_2 10 min	Andrade et al. protocol	based on personal communication with Andrade:<br>500-590 nm SYTO9<br>560-680 nm PI	10x	coronal	percentage of red fluorescence in distances:50-150 µm, 150-250 µm, 250-350 µm, 350-450 µm, 450-550 µm, 550-650 µm, 650-750 µm, 750-850 µm, 850-950 µm	measured until 950 µm
